# Combining the ABL1 Kinase Inhibitor Ponatinib and the Histone Deacetylase Inhibitor Vorinostat: A Potential Treatment for BCR-ABL-Positive Leukemia

**DOI:** 10.1371/journal.pone.0089080

**Published:** 2014-02-28

**Authors:** Seiichi Okabe, Tetsuzo Tauchi, Shinya Kimura, Taira Maekawa, Toshihiko Kitahara, Yoko Tanaka, Kazuma Ohyashiki

**Affiliations:** 1 First Department of Internal Medicine, Tokyo Medical University, Tokyo, Japan; 2 Division of Hematology, Respiratory Medicine and Oncology, Department of Internal Medicine, Faculty of Medicine, Saga University, Saga, Japan; 3 Department of Transfusion Medicine and Cell Therapy, Kyoto University Hospital, Kyoto, Japan; Institut national de la santé et de la recherche médicale (INSERM), France

## Abstract

Resistance to imatinib (Gleevec®) in cancer cells is frequently because of acquired point mutations in the kinase domain of BCR-ABL. Ponatinib, also known as AP24534, is an oral multi-targeted tyrosine kinase inhibitor (TKI), and it has been investigated in a pivotal phase 2 clinical trial. The histone deacetylase inhibitor vorinostat (suberoylanilide hydroxamic acid) has been evaluated for its significant clinical activity in hematological malignancies. Thus, treatments combining ABL TKIs with additional drugs may be a promising strategy in the treatment of leukemia. In the current study, we analyzed the efficacy of ponatinib and vorinostat treatment by using BCR-ABL-positive cell lines. Treatment with ponatinib for 72 h inhibited cell growth and induced apoptosis in K562 cells in a dose-dependent manner. We found that ponatinib potently inhibited the growth of Ba/F3 cells ectopically expressing BCR-ABL T315I mutation. Upon BCR-ABL phosphorylation, Crk-L was decreased, and poly (ADP-ribose) polymerase (PARP) was activated in a dose-dependent manner. Combined treatment of Ba/F3 T315I mutant cells with vorinostat and ponatinib resulted in significantly increased cytotoxicity. Additionally, the intracellular signaling of ponatinib and vorinostat was examined. Caspase 3 and PARP activation increased after combination treatment with ponatinib and vorinostat. Moreover, an increase in the phosphorylation levels of γH2A.X was observed. Previously established ponatinib-resistant Ba/F3 cells were also resistant to imatinib, nilotinib, and dasatinib. We investigated the difference in the efficacy of ponatinib and vorinostat by using ponatinib-resistant Ba/F3 cells. Combined treatment of ponatinib-resistant cells with ponatinib and vorinostat caused a significant increase in cytotoxicity. Thus, combined administration of ponatinib and vorinostat may be a powerful strategy against BCR-ABL mutant cells and could enhance the cytotoxic effects of ponatinib in those BCR-ABL mutant cells.

## Introduction

Chronic myeloid leukemia (CML) is a myeloproliferative disorder of hematopoietic stem cells caused by the presence of the *BCR-ABL* oncogene in the so-called Philadelphia chromosome (Ph) [1]. The BCR-ABL fusion protein is a constitutively active tyrosine kinase and activator of downstream molecules such as Myc and signal transducer and activator of transcription (STAT) [2], [3]. BCR-ABL activity promotes the growth of leukemic cells and enhances malignant expansion of hematopoietic stem cells.

The clinical outcome for patients with CML is improved by imatinib mesylate (Gleevec®) [4]. Imatinib was the first ABL tyrosine kinase inhibitor (TKI) that was identified to reduce BCR-ABL kinase activity. In the International Randomized Study of Interferon and STI571 (IRIS) trial, imatinib treatment resulted in a high level of cytogenetic response. However, some patients developed resistance to imatinib, which could be attributed to point mutations in the kinase domain of BCR-ABL [5]. These BCR-ABL mutations directly impede contact between the BCR-ABL protein and imatinib. Recently, second-generation ABL TKIs dasatinib (Sprycel®) and nilotinib (Tasigna®) have been increasingly used for patients resistant to or intolerant of imatinib therapy, and have been approved for front line use in patients with chronic phase CML [6]. However, one point mutation, T315I, located in the gatekeeper region of the ATP-binding site, confers resistance to imatinib, dasatinib, and nilotinib [7]. Until now, no viable treatment options were available for patients in whom ABL TKIs fail because of the presence of T315I mutation. Thus, alternative strategies are required to improve the outcome of CML patients carrying the T315I mutation.

Ponatinib, also known as AP24534, is an oral, multi-targeted TKI. Ponatinib is effective at nanomolar levels against T315I and other point mutations [8], [9]. This TKI has been investigated in a pivotal phase 2 clinical trial in patients with resistant or intolerant CML and Ph-positive acute lymphoblastic leukemia [10].

Histone acetyltransferases and histone deacetylases (HDACs) function antagonistically to control histone acetylation [11]. HDACs regulate chromatin remodeling and are crucial in the epigenetic regulation of various genes. Abnormal activity or expression of HDACs has been found in a broad range of tumor types [12]. An HDAC inhibitor (HDACi) blocks the activity of specific HDACs. Preclinical data suggest a role for HDACi as a potential new treatment in several tumor types, including hematological malignancies [13].

In this study, we investigated ponatinib activity against Ph-positive leukemia cells carrying the T315I mutation. We also examined the efficacy of HDACi vorinostat in combination with ponatinib in various cell lines. This study also aimed to explore the molecular mechanism of ponatinib resistance by using BCR-ABL-expressing cell lines with point mutations. Furthermore, co-treatment with ponatinib and vorinostat suppressed growth in ABL TKI ponatinib-resistant clones.

## Materials and Methods

### Reagents and antibodies

Ponatinib was purchased from Shanghai Biochempartner Co., Ltd. (Shanghai, China). The HDAC inhibitor vorinostat (suberoylanilide hydroxamic acid) was provided by Merck & Co (New Jersey, NJ). Stock solutions of vorinostat and ponatinib were dissolved in dimethyl sulfoxide (DMSO) and subsequently diluted to the desired concentration in the growth medium. Anti-phospho Abl, anti-phospho Crk-L, anti-cleaved caspase 3, anti-poly (ADP-ribose) polymerase (PARP), and anti-acetyl-histone H4 antibodies were purchased from Cell Signaling (Beverly, MA). β-Tubulin and β-actin antibodies were provided by Santa Cruz Biotechnology (Dallas, TX). Other reagents were obtained from Sigma (St Louis, MI).

### Cell culture and mutagenesis

The human CML cell line K562 was obtained from American Type Culture Collection (ATCC; Manassas, VA). The BCR-ABL-positive cell line Ba/F3 BCR-ABL with wild-type and mutant Ba/F3 cells (T315I) was previously established [14]. These cells were maintained in RPMI1640 medium supplemented with 10% heat-inactivated fetal bovine serum containing 1% penicillin/streptomycin in a humidified incubator at 37°C. Ponatinib-resistant Ba/F3 cells were established previously [15].

### BCR-ABL mutation analysis

Genomic DNA was isolated using the DNeasy kit (Qiagen, Valencia, CA). Specific subregions of *BCR-ABL* cDNA were amplified by high-fidelity PCR from genomic DNA by using a Stratagene Autocycler (Robocycler Gradient 40). The primers used for the reactions were SH3-SH2-upper, 5′-CTCCAGACTGTCCACAGCATTCC-3′, and SH3-SH2-lower, 5′-CTGTCATCAACCTGCTCAGGC-3′; SH2-kinase-upper, 5′-CGTGAGAGTGAGAGCAGTCC-3′, and SH2-kinase-lower, 5′-GCAGCTCTCCTGGAGGTCCTCG-3′. The PCR products were sequenced and analyzed by SRL (Tokyo, Japan).

### Primary samples

This study was approved by the Institutional Review Board of Tokyo Medical University, and written informed consent was provided by all patients in accordance with the Declaration of Helsinki. Primary samples were obtained from the peripheral blood samples of CML patients after consent. Mononuclear cells were separated by Lymphosepar (Immuno-Biological Laboratories Co., Gunma, Japan) and cultured in RPMI1640 medium.

### Cell proliferation assay

Cell proliferation assay was performed as previously described [16]. Briefly, cells were plated in 96-well cell culture plates (8×10^3^ cells/well) and were treated with ponatinib and/or vorinostat at the indicated concentrations. Treated cells were stained with a cell counting kit solution (Dojin, Kumamoto, Japan) and measured photometrically (450 nm) to determine cell viability. All experiments were performed three times.

### Immunoblot analysis

Immunoblot analysis was performed as previously described [17], [18]. In brief, after treatment with ponatinib and/or vorinostat, the protein contents of the lysates were determined with a protein assay kit (Bio-Rad Laboratories, Hercules, CA). Proteins were loaded onto polyacrylamide gels and then transferred to polyvinylidene difluoride membranes (Millipore, Bedford, MA). The membranes were incubated with the primary antibodies of interest at the appropriate dilution for 1 h. Blots were then probed with secondary antibodies and developed using the enhanced chemiluminescence system (ECL, Amersham Phamacia Biotech, Bucks, UK).

### In vivo assays

Nude mice were purchased from CLEA Japan, Inc. (Tokyo, Japan). The mice (n = 5) were injected subcutaneously with 1×10^7^ Ba/F3 T315I mutant cells. This study was carried out in strict accordance with the recommendations in the Guide for the Care and Use of Laboratory Animals of the Tokyo Medical University. The protocol was also approved by the Committee on the Ethics of Animal Experiments of the Tokyo Medical University (Permit Number: H-24023). All animal care complied with institutional guidelines. The mice were treated orally with 30 mg/kg ponatinib or 70 mg/kg vorinostat intraperitoneally, or with a combination of the two agents five days per week. Control mice were treated with 0.9% NaCl of the vehicle for ponatinib. The animals were examined every week for the development of tumors. Xenografts were measured three times per week, and tumor volumes were calculated (L×W×H/2). After euthanasia, tissue was excised, fixed in 10% formalin, and prepared for standard histopathological examination.

### Statistical analysis

Differences between treatment groups, in terms of dose response and apoptosis, were determined using the Welch's *t* test, accounting for unequal variance. P values<0.05 were considered significant. In some experiments, data for comparison of multiple groups are presented as mean ± S.D. and analyzed with two-way ANOVA.

## Results

### Ponatinib inhibits growth and induces apoptosis in K562 and T315I mutant cells

**Figure 1:**
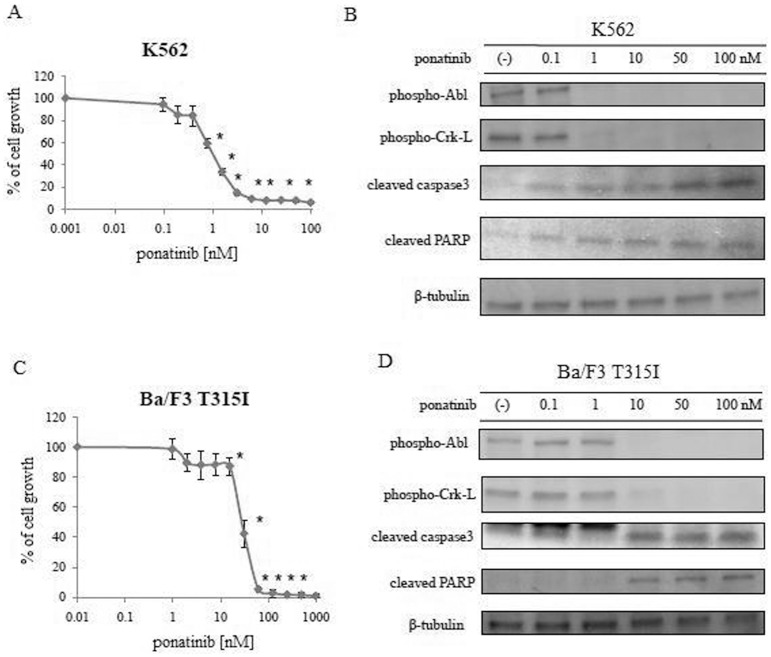
Effect of ponatinib on BCR-ABL-expressing cells. (A) K562 cells were cultured at a concentration of 8×10^4^/mL in the presence of varying concentrations of ponatinib for 72 h. The number of viable cells was calculated. Results are representative of three separate experiments. *P<0.05, ponatinib treatment versus control. (B) K562 cells were treated with ponatinib for 24 h, and total extracts were examined by immunoblot analysis with anti-phospho ABL, anti-Crk-L, anti-cleaved caspase 3, anti-PARP, and anti-β-tubulin antibodies. (C) Ba/F3 T315I cells were cultured in the presence of varying concentrations of ponatinib for 72 h. The number of viable cells was calculated. Results are representative of three separate experiments. *P<0.05, ponatinib treatment versus control. (D) Ba/F3 T315I cells were treated with ponatinib for 24 h, and total extracts were examined by immunoblot analysis with anti-phospho ABL, anti-Crk-L, anti-cleaved caspase 3, anti-PARP, and anti-tubulin antibodies.

We evaluated the efficacy of ponatinib in a representative BCR-ABL-expressing cell line, namely, K562. K562 cells were treated with varying concentrations of ponatinib for 72 h. Treatment with ponatinib for 72 h significantly reduced growth of K562 cells at nanomolar levels (Figure 1A). We also examined the intracellular signaling of ponatinib. Immunoblots showed that phosphorylation of both BCR-ABL and its downstream molecule Crk-L was significantly reduced after treatment with ponatinib. In addition, caspase 3 and PARP activity was increased by ponatinib in a dose-dependent manner (Figure 1B). It has been reported that ponatinib has potent activity against T315I mutant cells [8]. Thus, we examined ponatinib activity by using Ba/F3 BCR-ABL T315I mutant cells. In concordance with previously reported results, ponatinib inhibited growth in Ba/F3 T315I cells at low concentrations (Figure 1C). Immunoblot analysis revealed that this inhibition was caused by reduced phosphorylation of BCR-ABL and Crk-L and increased activation of PARP in Ba/F3 BCR-ABL T315I cells after treatment with ponatinib (Figure 1D).

### Efficacy of ponatinib and HDACi against T315I mutant cells

**Figure 2:**
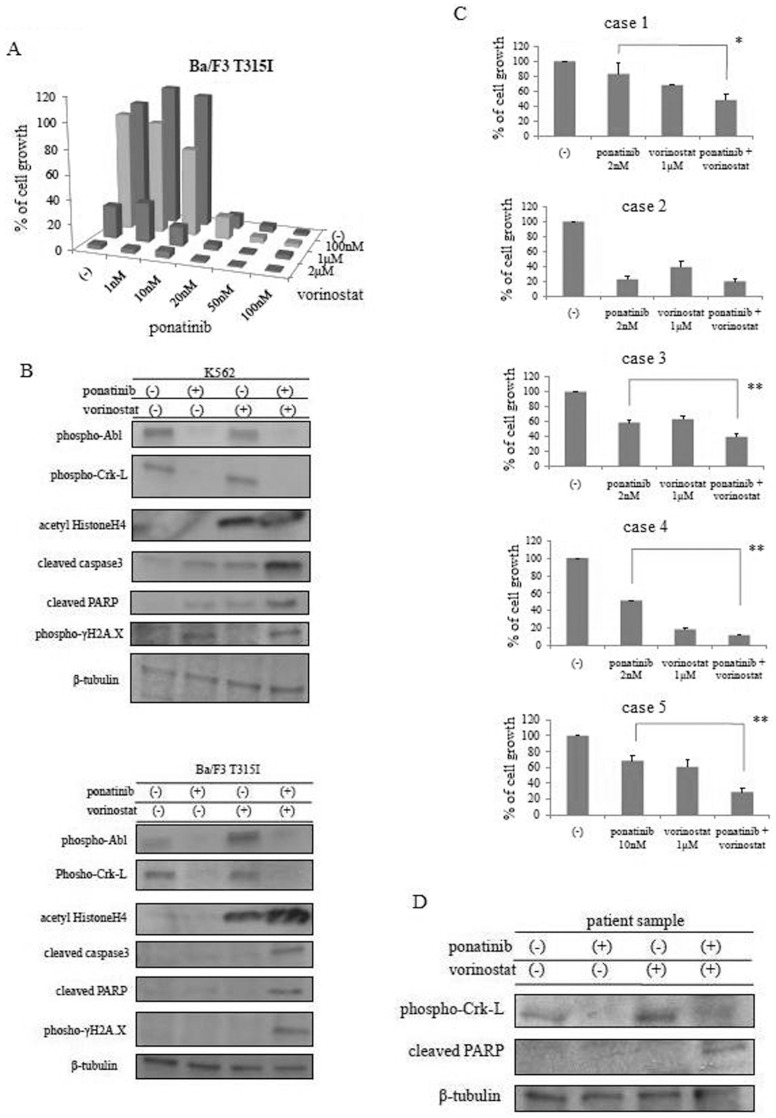
Ponatinib and vorinostat increase cell growth inhibition and induce apoptosis in BCR-ABL-expressing cells. (A) Cells were cultured at a concentration of 8×10^4^/mL in the presence or absence of ponatinib and/or vorinostat for 72 h. The percentage of proliferating cells was evaluated, as described in Materials and Methods. (B) K562 or Ba/F3 T315I cells were treated with ponatinib and/or vorinostat for 24 h, and total extracts were examined by immunoblot analysis with anti-phospho ABL, Crk-L, γH2A.X, cleaved caspase 3, PARP, acetyl histone H4, and tubulin antibodies. (C) Primary samples were cultured in the presence or absence of ponatinib and/or vorinostat. The number of living cells was calculated. *P<0.05 or **P<0.01, ponatinib and vorinostat treatment versus ponatinib treatment. (D) Primary cells were treated with ponatinib and/or vorinostat at the indicated concentrations for 24 h. The cells were examined by immunoblot analysis.

HDACs belong to a family of enzymes involved in the deacetylation of lysine residues on histone and non-histone proteins. HDACis represent a new class of antitumor agents, and several HDACis have been investigated in hematological malignancies. We previously reported that an HDACi, depsipeptide, is effective against BCR-ABL-positive leukemia cells [19]. Therefore, we examined the activity of an HDACi against T315I mutant cells. We found that another HDACi, vorinostat, was effective against Ba/F3 T315I cells (Figure S1). We examined whether treatment with ponatinib and vorinostat induced cell death in T315I mutant cells. We found that treatment with ponatinib and vorinostat significantly inhibited T315I mutant cell growth compared to treatment with single drugs (Figure 2A). Two-way ANOVA analysis revealed that the combined effects of the drugs was synergistic. We also investigated intracellular signaling and found that after BCR-ABL phosphorylation, Crk-L activity reduced, PARP cleaved, and γH2A.X phosphorylation increased after ponatinib and vorinostat treatment (Figure 2B). We next examined ponatinib and vorinostat co-treatment in primary Ph+ leukemia samples. The clinical data for the CML- and Ph-positive leukemia patients are documented (Table S1). Analysis of whole blood cells with BCR-ABL fluorescence in situ hybridization showed that >95% of cells were BCR-ABL positive. When primary cell samples were treated with a combination of ponatinib and vorinostat, cell growth was significantly inhibited compared to the untreated samples (Figure 2C). Immunoblot analysis revealed that Crk-L phosphorylation reduced and PARP activity increased after ponatinib and vorinostat co-treatment (Figure 2D).

### Effects of ponatinib and HDACi on T315I mutant cells in a xenograft model

**Figure 3:**
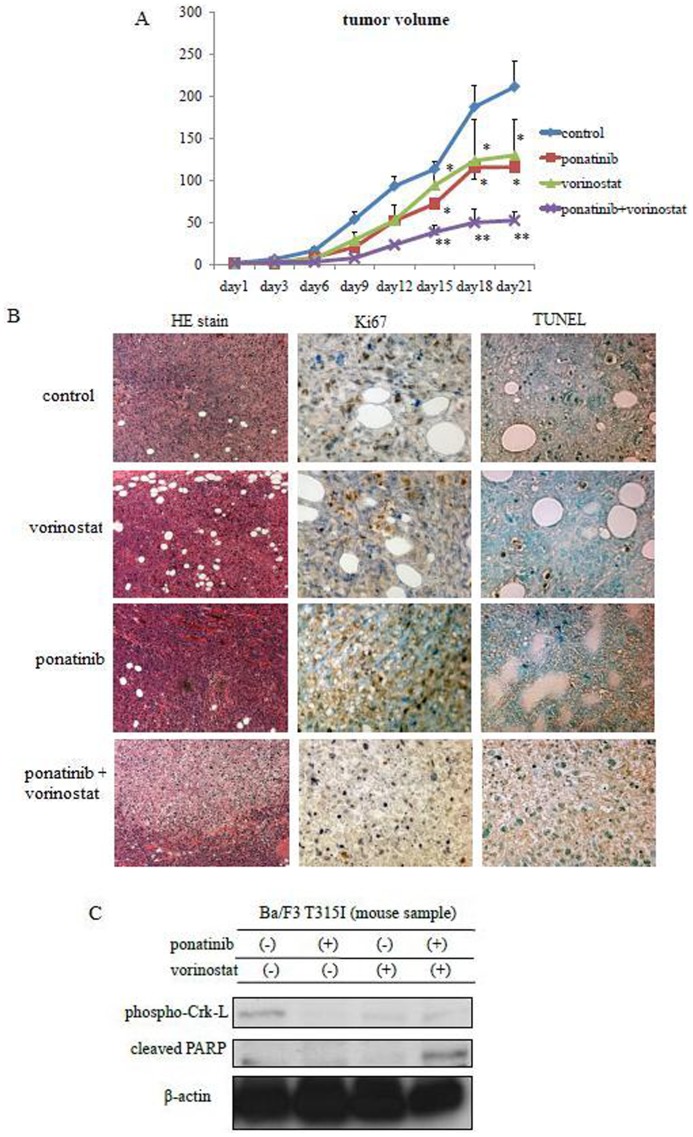
Effects of ponatinib and vorinostat on Ba/F3 T315I cells in a xenograft model. (A) *In vivo* studies were performed as described in Materials and Methods. At various time points, tumor sizes were reported. Average tumor weight per mouse was calculated and used to calculate the group mean tumor weight ± s.d (n = 5). (B) Immunohistochemical staining for hematoxylin and eosin (HE) at a magnification of 40×, Ki67 (magnification, 400×), and TUNEL (magnification, 400×) in Ba/F3 T315I xenograft sections. (C) Tumor cells treated with or without ponatinib and vorinostat were examined by immunoblot analysis.

To confirm the effect of ponatinib and vorinostat on T315I mutant cells, we examined their activity in a mouse xenograft model. Nude mice were injected subcutaneously with 1×10^7^ Ba/F3 T315I mutant cells, and tumor volumes were evaluated every three days. We observed that the growth of tumors after treatment with ponatinib or vorinostat was partially reduced. In comparison, co-treatment with ponatinib and vorinostat significantly reduced tumor growth (Figure 3A). Upon immunohistochemical staining, Ki67, a marker of cellular proliferation, was significantly reduced in case of co-treatment with ponatinib and vorinostat compared to the control. In TdT-mediated dUTP nick-end labeling (TUNEL) staining, the number of apoptotic cells in the tumor sections of the group treated with ponatinib and vorinostat was higher than in those of the control group (Figure 3B). Thus, co-treatment with ponatinib and vorinostat inhibited tumor growth and induced apoptosis in T315I-positive Ba/F3 cells in the xenograft. We next investigated the intracellular signaling in a xenograft protein extract. Crk-L phosphorylation reduced and PARP activity increased after co-treatment with ponatinib and vorinostat. These results indicated that co-treatment with ponatinib and vorinostat was effective against T315I mutant cells in the xenograft model (Figure 3C).

### Analysis of ABL TKI resistant Ba/F3 BCR-ABL cells

**Figure 4:**
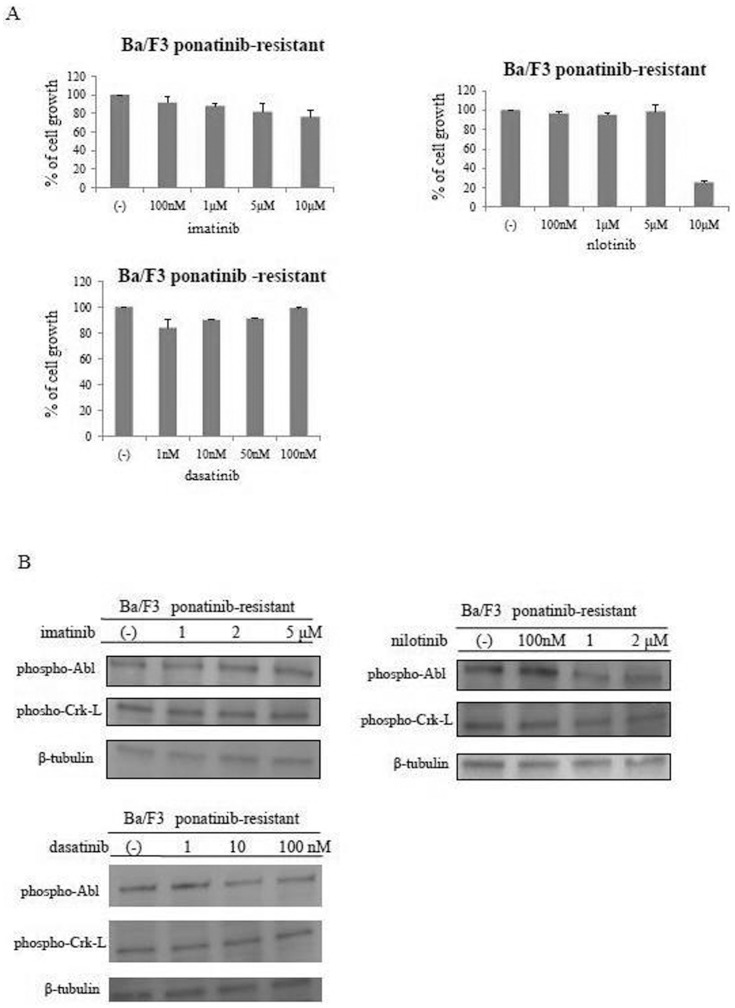
Analysis of ponatinib-resistant Ba/F3 BCR-ABL cells. (A) Ba/F3 ponatinib-resistant cells were treated with various concentrations of imatinib, nilotinib, or dasatinib, and cellular growth was analyzed. These experiments were performed in triplicate. (B) Ba/F3 ponatinib-resistant cells were treated with imatinib, nilotinib, or dasatinib for 24 h. Whole cell lysates were analyzed by immunoblotting with phospho-specific Abl and Crk-L Abs. β-tubulin was used as the loading control.

Ponatinib-resistant Ba/F3 (AP-R) cells were established previously [15]. After performing a cell growth assay using additional ABL TKIs (e.g., imatinib, dasatinib, and nilotinib), we found that Ba/F3 AP-R cells were highly resistant to imatinib, dasatinib, and nilotinib, as well (Figure 4A). We investigated intracellular signaling by using Ba/F3 AP-R cells. We observed that BCR-ABL and Crk-L phosphorylation was not reduced by treatment with imatinib, dasatinib, or nilotinib (Figure 4B).

### Co-treatment with ponatinib and vorinostat is effective against and induces apoptosis in ponatinib-resistant cells

**Figure 5:**
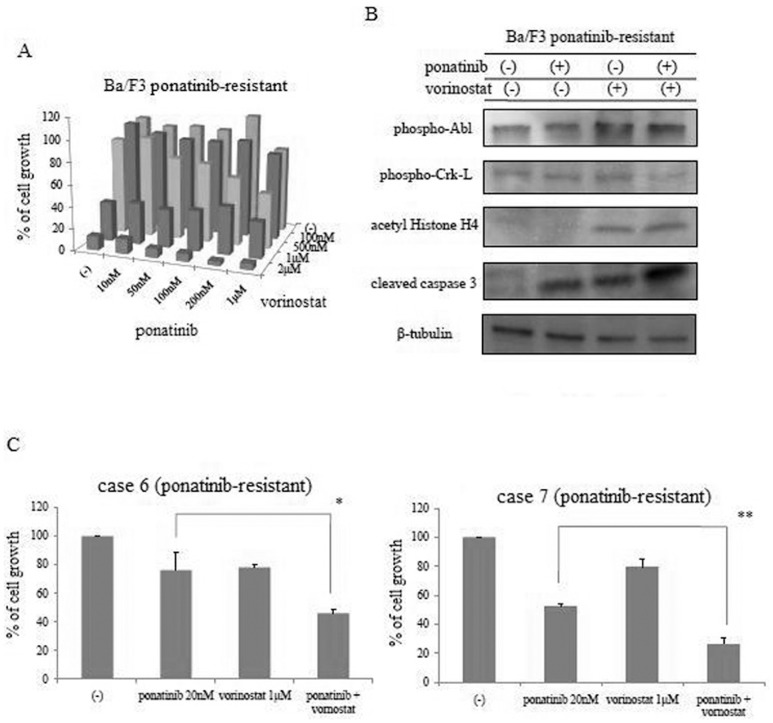
Effects of ponatinib and vorinostat on ABL TKI-resistant cells. (A) Ba/F3 AP-R cells were cultured in the presence or absence of ponatinib and/or vorinostat and the viable cells were enumerated. (B) Ba/F3 AP-R cells were treated with the indicated concentrations of ponatinib and/or vorinostat for 24 h. Total cell extracts were analyzed by immunoblotting with phospho-specific Abl, anti-Crk-L, and anti-cleaved PARP antibodies. β-Actin was used as loading control. The results in A and B are representative of at least three reproducible experiments. (C) Ponatinib-resistant primary samples were cultured in the presence or absence of ponatinib and/or vorinostat and the viable cells were enumerated. *P<0.05 or **P<0.01, ponatinib and vorinostat treatment versus ponatinib treatment.

Since vorinostat was effective against T315I mutant cells, we investigated whether ponatinib-resistant cells were inhibited by this HDACi. We observed that growth of Ba/F3 ponatinib-resistant cells was significantly reduced by vorinostat in a dose-dependent manner (data not shown). We also examined the efficacy of combined treatment with ponatinib and vorinostat against ponatinib-resistant cells. Combined treatment with ponatinib and vorinostat significantly reduced the growth of Ba/F3 ponatinib-resistant cells (Figure 5A). We also found that Crk-L phosphorylation reduced and caspase 3 activity increased after ponatinib and vorinostat co-treatment (Figure 5B). Furthermore, we examined the efficacy of this treatment in ponatinib-resistant primary Ph-positive acute lymphoblastic leukemia samples and found that ponatinib and vorinostat in combination significantly reduced the cellular growth of ponatinib-resistant primary samples (Figure 5C). These results indicate that co-treatment with ponatinib and vorinostat may be effective against ABL TKI-resistant BCR-ABL cells.

## Discussion

Ponatinib is effective against T315I mutant cells that are resistant to imatinib and second-generation ABL TKIs nilotinib and dasatinib. O'Hare and colleagues reported that treatment with 40 nM ponatinib did not yield any BCR-ABL mutant cells [8]. We confirmed that ponatinib was effective against BCR-ABL wild-type and T315I mutant cells at low concentrations by cell proliferation and immunoblot assays.

An important finding in this study was that combined treatment with ponatinib and vorinostat showed antiproliferative effects *in vitro* and exhibited antitumor activity *in vivo*. Using the Ba/F3 T315I xenograft model, ponatinib or vorinostat (30 and 70 mg/kg, respectively) showed similar reduction in tumor size. We demonstrated the tumor volumes in mice treated with both ponatinib and vorinostat were significantly reduced compared to those treated with each drug alone. Immunohistochemical analysis revealed that the expression of the proliferation marker Ki67 reduced and TUNEL-positive cells increased in ponatinib and vorinostat-treated mice. These results suggest that this combination was effective against T315I mutation *in vivo*. Overall, the results indicate that a greater level of efficacy was achieved with combined treatment with ponatinib and vorinostat.

Multiple preclinical studies and clinical data support the use of HDACis in combination with other drugs for the treatment of various cancers, including leukemia. Some HDACis, including vorinostat and romidepsin, have been approved for use against cutaneous T-cell lymphoma [20], [21]. HDACis have multiple biological effects related to acetylation of histone and non-histone proteins, such as the chaperone heat shock protein 90 (HSP90) [22]. Vorinostat induces HSP90 hyperacetylation and inhibits its chaperone function. Thus, vorinostat may inhibit the growth of BCR-ABL-positive cells by changing BCR-ABL conformation via acetylation and inhibition of the chaperone protein HSP90.

Phosphorylated γH2A.X is associated with early DNA damage and repair processes that occur in response to double-strand breaks in eukaryotic cells [23]. Vorinostat induced growth arrest and apoptosis, thus aggravating the apoptotic and cytotoxic effects of ponatinib on Ba/F3 T315I mutant cells. Since imatinib inhibits STAT5 phosphorylation as well as the expression of STAT5 target genes [24], [25], ponatinib may exhibit the same inhibitory effect. In our immunoblot assay, γH2A.X phosphorylation was detected after co-treatment with ponatinib and vorinostat. Co-treatment with ponatinib and vorinostat resulted in increased cytotoxicity and provided strong evidence that vorinostat augments ponatinib-induced apoptosis by enhancing DNA damage responses in BCR-ABL-positive cells.

Patients with hematological malignancies, including Ph-positive leukemia, often develop resistance to TKIs. In our study, we used Ba/F3 AP-R BCR-ABL cells and primary samples. We demonstrated that co-treatment with ponatinib and vorinostat reduced the proliferation of ponatinib-resistant cells. Therefore, ponatinib and vorinostat may affect the activity of BCR-ABL and increase antileukemic activity against BCR-ABL mutant cells. Recently, the use of ponatinib has been evaluated in other hematological malignancies and its use has been approved by the FDA [26]. We previously isolated primary cells highly resistant to ponatinib showing several BCR-ABL point mutations [15]. Thus, ponatinib resistance seems to be a possible concern in near future, and therefore, methods to overcome ABL TKI resistance need to be developed.

In summary, our results provide new information on the molecular events underlying the antitumor activity of ponatinib and the HDAC inhibitor vorinostat. Co-treatment using these compounds together with molecular-targeted drugs will benefit those with BCR-ABL leukemic cells that are resistant to conventional treatments.

## Supporting Information

Figure S1 Effect of vorinostat on Ba/F3 T315I cells. Ba/F3 T315I cells were cultured at a concentration of 8×10^4^/mL in the presence of varying concentrations of vorinostat for 72 h and viable cells were enumerated. Results are representative of three separate experiments. *P<0.05, vorinostat treatment versus control.
(TIF)

Table S1 Summary of CML chronic-phase and Ph+ ALL patient samples. Primary samples were collected before treatment except for samples noted in the table that received treatment with tyrosine kinase inhibitor therapy with imatinib or ponatinib and used in the cell proliferation and immunoblot analysis. N/A, data not available.
(TIF)
