# Supplementary figures and images for: Combining the ABL1 Kinase Inhibitor Ponatinib and the Histone Deacetylase Inhibitor Vorinostat: A Potential Treatment for BCR-ABL-Positive Leukemia
Source: PLoS One. 2014 Feb 28;9(2):e89080. doi: 10.1371/journal.pone.0089080 (PMC3938434; doi:10.1371/journal.pone.0089080)

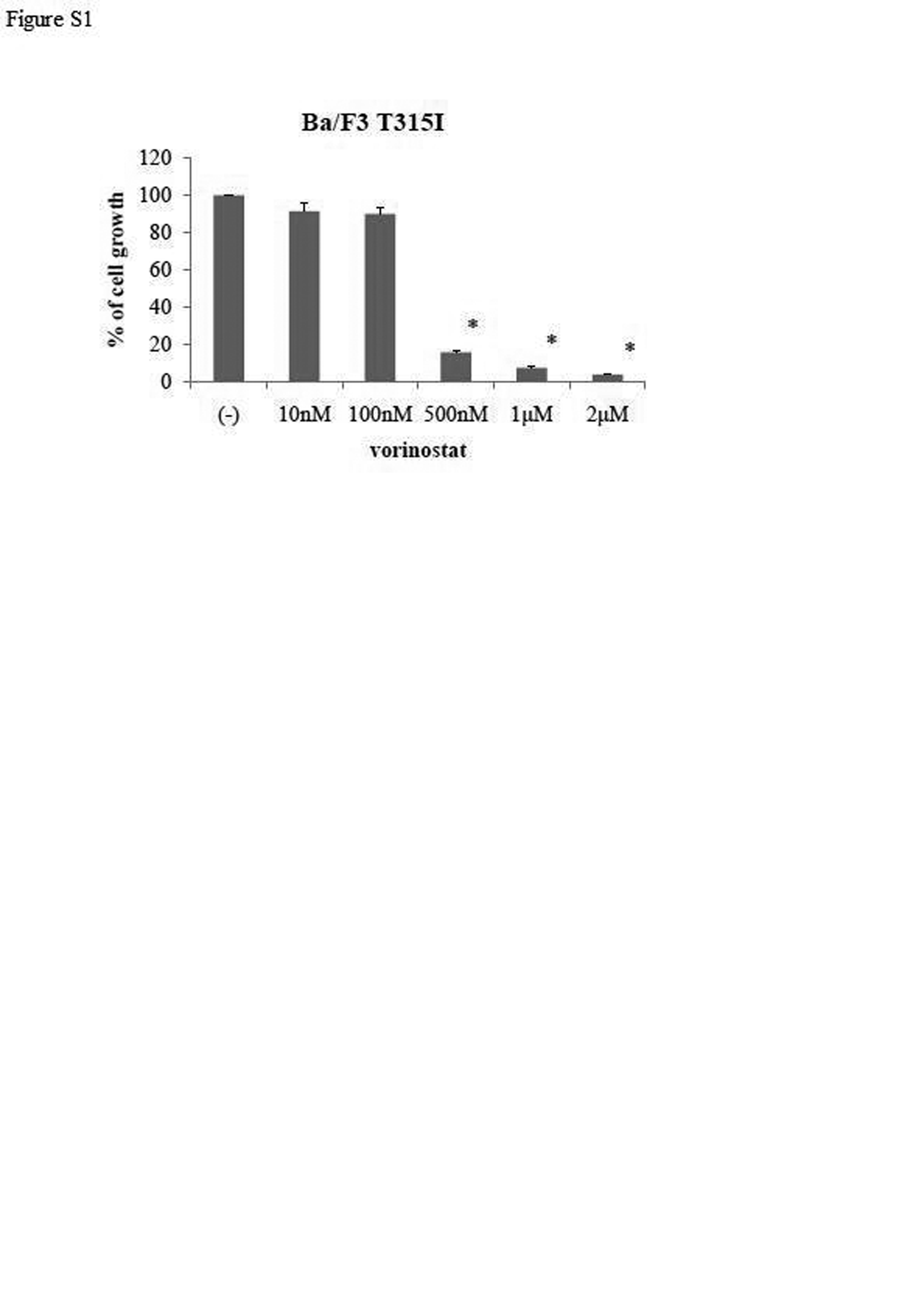

Supplement: Figure S1 — Effect of vorinostat on Ba/F3 T315I cells. Ba/F3 T315I cells were cultured at a concentration of 8×10^4^/mL in the presence of varying concentrations of vorinostat for 72 h and viable cells were enumerated. Results are representative of three separate experiments. *P<0.05, vorinostat treatment versus control. (TIF) [file pone.0089080.s001.tif]

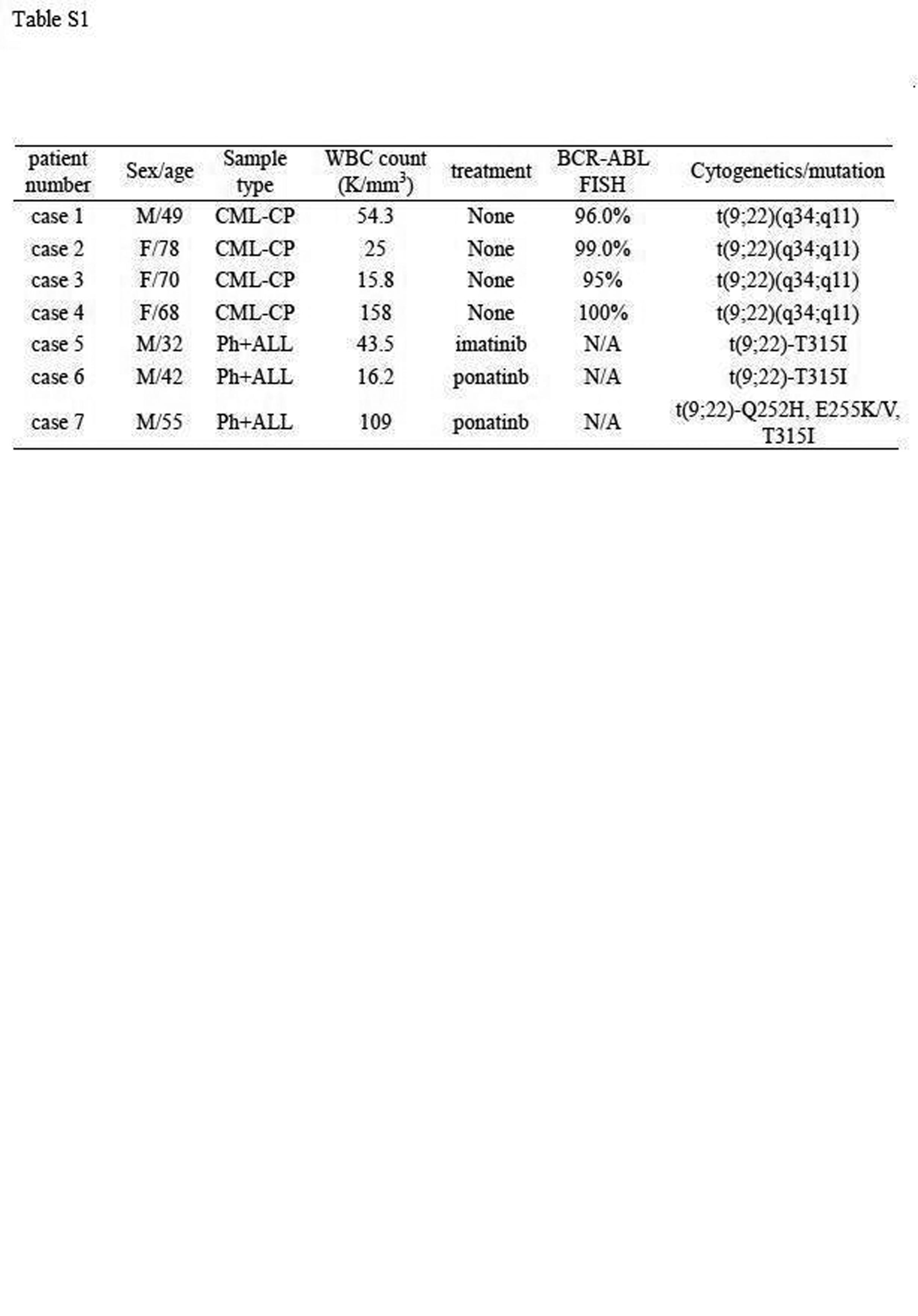

Supplement: Table S1 — Summary of CML chronic-phase and Ph+ ALL patient samples. Primary samples were collected before treatment except for samples noted in the table that received treatment with tyrosine kinase inhibitor therapy with imatinib or ponatinib and used in the cell proliferation and immunoblot analysis. N/A, data not available. (TIF) [file pone.0089080.s002.tif]
